# Normal Neurochemistry in the Prefrontal and Cerebellar Brain of Adults with Attention-Deficit Hyperactivity Disorder

**DOI:** 10.3389/fnbeh.2015.00242

**Published:** 2015-09-28

**Authors:** Dominique Endres, Evgeniy Perlov, Simon Maier, Bernd Feige, Kathrin Nickel, Peter Goll, Emanuel Bubl, Thomas Lange, Volkmar Glauche, Erika Graf, Dieter Ebert, Esther Sobanski, Alexandra Philipsen, Ludger Tebartz van Elst

**Affiliations:** ^1^Section for Experimental Neuropsychiatry, Department for Psychiatry and Psychotherapy, University Medical Center Freiburg, Freiburg, Germany; ^2^Department of Radiology, Medical Physics, University Medical Center Freiburg, Freiburg, Germany; ^3^Freiburg Institute for Advanced Studies, Albert-Ludwigs-University, Freiburg, Germany; ^4^Department of Neurology, University Medical Center Freiburg, Freiburg, Germany; ^5^Clinical Trials Unit, University Medical Center Freiburg, Freiburg, Germany; ^6^Clinic for Psychiatry and Psychotherapy, Central Institute for Mental Health Mannheim, Mannheim, Germany

**Keywords:** attention-deficit hyperactivity disorder, MRS, glutamate, anterior cingulate cortex, cerebellum, nosology

## Abstract

Attention-deficit hyperactivity disorder (ADHD) is a common neurodevelopmental disorder. In an attempt to extend earlier neurochemical findings, we organized a magnetic resonance spectroscopy (MRS) study as part of a large, government-funded, prospective, randomized, multicenter clinical trial comparing the effectiveness of specific psychotherapy with counseling and stimulant treatment with placebo treatment (Comparison of Methylphenidate and Psychotherapy Study). We report the baseline neurochemical data for the anterior cingulate cortex (ACC) and the cerebellum in a case–control setting. For the trial, 1,480 adult patients were contacted for participation, 518 were assessed for eligibility, 433 were randomized, and 187 were potentially eligible for neuroimaging. The control group included 119 healthy volunteers. Single-voxel proton MRS was performed. In the patient group, 113 ACC and 104 cerebellar spectra fulfilled all quality criteria for inclusion in statistical calculations, as did 82 ACC and 78 cerebellar spectra in the control group. We did not find any significant neurometabolic differences between the ADHD and control group in the ACC (Wilks’ lambda test: *p* = 0.97) or in the cerebellum (*p* = 0.62). Thus, we were unable to replicate earlier findings in this methodologically sophisticated study. We discuss our findings in the context of a comprehensive review of other MRS studies on ADHD and a somewhat skeptical neuropsychiatric research perspective. As in other neuropsychiatric disorders, the unclear nosological status of ADHD might be an explanation for false-negative findings.

## Introduction

Attention-deficit hyperactivity disorder (ADHD) is a common and often debilitating disorder that has received increasing public and scientific attention. In adulthood, the prevalence rates are estimated at 2–4% (Biederman, 2005; Philipsen et al., 2008). According to the criteria outlined in the *Diagnostic and Statistical Manual of Mental Disorders, Fifth Edition* (DSM-5), there are three presentations of ADHD: the inattentive subtype (iADHD), the hyperactive-impulsive subtype (hADHD), and the combined subtype (cADHD).^1^ The dopaminergic system seems to play a central role in the pathophysiology of ADHD (Philipsen et al., 2008). Treatment options for adult ADHD include pharmacological interventions (Castells et al., 2011) and psychotherapy (Philipsen, 2012). However, because evidence of the efficacy of these methods in adults is sparse, the treatment options require further investigation (Volkow and Swanson, 2013).

### The COMPAS study

In order to investigate treatment options for adult ADHD, the Comparison of Methylphenidate and Psychotherapy Study (hereafter the COMPAS study), a prospective, double-blind, placebo-controlled, multicenter clinical trial, was funded by the German Federal Ministry of Education and Research (BMBF: ADHD-NET: 01GV0605, 01GV0606) between 2006 and 2013. Using a factorial four-arm design, the COMPAS study compared the efficacy of cognitive behavioral group psychotherapy to that of clinical management in combination with methylphenidate (MPH) or placebo (Philipsen, 2008). As an integral part of the COMPAS study in the present magnetic resonance spectroscopy (MRS) study, we aimed to assess the neurochemical neuronal health of enrolled patients and to obtain evidence of putative dopaminergic dysfunction in measuring glutamate signals without exposure to radiation.

### Magnetic resonance spectroscopy

Magnetic resonance spectroscopy is a unique, non-invasive method of measuring different metabolites in the human brain. Single-voxel spectroscopy (SVS) is the most established method and allows an absolute quantification of the following neurometabolites: glutamate (Glu) and glutamine (Gln) and the combined Glu and Gln signal (Glx); phosphocholine (PCh) and glycerophosphorylcholine (GPC) and the combined total choline signal (t-Cho); and *N*-acetylaspartate (NAA), creatine (Cre), and myo-Inositol (mI). Glu is the major excitatory neurotransmitter in the human brain. Gln is the precursor and storage form of Glu in astrocytes. The activity of the glutamate system is closely interwoven with dopaminergic neurotransmission. NAA is regarded as a marker of overall neuronal and axonal integrity. t-Cho indicates cell-membrane turnover. Cre is a marker of brain energy metabolism. Lastly, mI is a glial marker as well as a part of the phosphatidylinositol second messenger system (Ross and Bluml, 2001). Since MRS is capable of quantifying all of these neurometabolites under specific conditions, it could provide a comprehensive assessment of the neurochemical health of the brain (Perlov et al., 2009).

### Previous MRS findings regarding ADHD

We performed a comprehensive review of the MRS literature on ADHD on the basis of a PubMed search using the search terms “ADHD” and “spectroscopy.” We identified 416 hits (on May 30, 2015) that were individually screened for content. Excluding methodological papers, reviews, and case reports, we identified 32 MRS studies on ADHD. All of these studies are presented in Table 1, and we discuss this literature in detail in the discussion section of the paper. During the finalization of our study protocol (2006), there was first evidence – albeit limited – of altered fronto-striatal NAA signals (Hesslinger et al., 2001; Jin et al., 2001) and increased glutamate signals (MacMaster et al., 2003; Courvoisie et al., 2004), which were reduced under medication with stimulants (Carrey et al., 2002, 2003).

**Table 1:** **Summary of previous MRS studies on ADHD**.

Study	Population	*n* (ADHD/Controls)	Methods	Region(s)	Results
1. Hesslinger et al. (2001)	Adults; iADHD, cADHD	10/5	2 T, SVS, 1H-MRS (PRESS)	DLPFC le Striatum le	NAA ↓ in cADHD vs. control/iADHD ↔
2. Jin et al. (2001)	Children	12/10	1.9 T, SVS, 1H-MRS (PRESS); MPH once	Striatum le Striatum ri	NAA/Cre ↓ NAA/Cre ↓, t-Cho/Cre ↑; MPH-once without effect
3. Carrey et al. (2002)	Children	4/0	1.5 T, SVS, 1H-MRS (PRESS); before and after MPH/AM	Striatum le PFC ri	Glx/Cre ↓ under MPH + AM Glx/Cr ↓ only under AM
4. MacMaster et al. (2003)	Children	9/9	1.5 T, SVS, 1H-MRS (PRESS)	Striatum le PFC ri	↔ Glx/Cre ↑
5. Carrey et al. (2003)	Children; iADHD, cADHD	14/0	1.5 T, SVS, 1H-MRS (PRESS); before and after MPH (4)/AM (3)/Dexedrine (7)	Striatum le PFC ri	Glx/Cre ↓ under medication ↔
6. Yeo et al. (2003)	Children; hADHD, iADHD	23/24	1.5 T, SVS, 1H-MRS (STEAM)	Frontal ri	↔
7. Courvoisie et al. (2004)	Children; hADHD	8/8	1.5 T, SVS, 1H-MRS (STEAM)	Frontal ri Frontal le	Glu/Cre ↑, NAA/Cre ↑, t-Cho/Cre ↑ Glu/Cre ↑
8. Sparkes et al. (2004)	Children	8/6	1.5 T, SVS, 1H-MRS (PRESS)	Striatum le PFC ri	↔ ↔
9. Fayed and Modrego (2005)	Children	8/12	1.5 T, SVS, 1H-MRS (PRESS)	Centrum semiovale le	NAA/Cre ↑
10. Sun et al. (2005)	Children; iADHD, cADHD	20/10	1.9 T, SVS, 1H-MRS (PRESS)	Nucleus lenticularis ri Nucleus lenticularis le	NAA/Cre ↓ in cADHD (vs. iADHD and controls) NAA/Cre ↓ in cADHD (vs. controls)
**11. Moore et al. (2006)**	**Children**	**15 (**+**8 patients with comorbid bipolar disorder)/7**	**1.5 T, SVS, 1H-MRS (STEAM)**	**ACC**	**Glx/mI** ↑ **(vs. controls/comorbid bipolar disorder), Glx/Cre** ↑ **(vs. comorbid bipolar disorder)**
12. Stanley et al. (2006)	Children; iADHD, cADHD	10/15	1.5 T, multivoxel 31-P-MRS	PFC Basal ganglia	Free-PME ↓ Free-PME ↓, free-PME/free-PDE ↓
				Superior temporal	↔
13. Fayed et al. (2007)	Children	22/8	1.5 T, SVS, 1H-MRS (PRESS)	Centrum semiovale le PFC ri	NAA/Cre ↑ NAA/Cre ↑
14. Carrey et al. (2007)	Children; cADHD	13/10	1.5 T, SVS, 1H-MRS (PRESS); before and after MPH	Striatum le PFC ri Occipital lobe	Glx ↑, Cre ↑; Cre ↓ after MPH ↔ ↔
**15. Perlov et al. (2007)**	**Adults**	**28/28**	**1.5 T, CSI, 1H-MRS (PRESS)**	**ACC ri** **ACC le**	**Glx/Cre** ↓ **↔**
**16. Colla et al. (2008)**	**Adults**	**15/10**	**1.5 T, CSI, 1H-MRS (PRESS)**	**ACC both sides**	**t-Cho** ↑
17. Stanley et al. (2008)	Children; iADHD, cADHD	31/36	1.5 T, multivoxel 31-P-MRS	Basal ganglia Inferior parietal Superior temporal Posterior white matter Occipital lobe	free-PME ↓ Free-PME ↑, free-PME/free-PDE ↑ ↔ ↔ ↔
**18. Kronenberg et al. (2008)**	**Adults**	**7/0**	**1.5 T, CSI, 1H-MRS (PRESS); before and after MPH**	**ACC both sides**	**t-Cho** ↓**, NAA** ↑ **after MPH**
19. Ferreira et al. (2009)	Adults; iADHD, cADHD	19/12	1.5 T, SVS, 1H-MRS (PRESS)	VMPFC ri VMPFC le Thalamus ri Thalamus le Striatum ri Striatum le	mI/Cre ↓ (in cADHD vs. controls) ↔ ↔ t-Cho/Cre ↑ (in cADHD vs. iADHD) ↔ Glx/Cre ↑ (in cADHD vs. iADHD/controls)
20. Yang et al. (2010)	Children; cADHD, iADHD	15/22	1.5 T, SVS, 1H-MRS (PRESS)	PFC ri PFC le	Cre ↓, NAA/Cr ↑ ↔
**21. Soliva et al. (2010)**	**Children; iADHD, hADHD, cADHD**	**17/17**	**1.5 T, SVS, 1H-MRS (PRESS)**	**DLPFC ri** **Cerebellum le**	**Cre** ↓ **mI** ↓**, NAA** ↓**, Cre** ↓
**22. Perlov et al. (2010)**	**Adults; cADHD**	**30/30**	**1.5 T, CSI, 1H-MRS (PRESS)**	**Cerebellum le** **Cerebellum ri** **Cerebellar vermis**	**Glx/Cre** ↑ ↔ ↔
**23. Dramsdahl et al. (2011)**	Adults; hADHD, iADHD, cADHD	29/38	3 T, SVS, 1H-MRS (PRESS)	Frontal le Frontal ri	Glu/Cre ↓ ↔
24. Hammerness et al. (2012)	**Children**	**10/12**	**4 T, SVS, 1H-MRS (PRESS); before and after MPH**	**ACC both sides**	↔**, no significant changes under MPH**
25. Edden et al. (2012)	Children; iADHD, cADHD	13/19	3T, SVS, 1H-MRS (PRESS)	Motor cortex le	GABA↓
**26. Wiguna et al. (2012)**	**Children; iADHD, cADHD**	**21/0**	**1.5 T, SVS, 1H-MRS; before and after MPH**	**PFC le** **PFC ri**	**NAA/Cre** ↑**, Glu/Cre** ↓**, t-Cho/Cre** ↓**, mI/Cr** ↓ **after MPH** **NAA/Cre** ↑**, Glu/Cre** ↓**, t-Cho/Cre** ↓ **after MPH**
**27. Arcos-Burgos et al. (2012)**	**Children, adults**	**14/20**	**1.5 T, CSI, 1H-MRS (PRESS)**	**Striatum ri, striatum le, corpus callosum (splenium), ACC ri, ACC le, MCC ri, MCC le, PCC ri, PCC le, thalamus ri (lateral and medial), thalamus le (lateral and medial), and cerebellar vermis**	**PCC ri showed significant differences of the Glx/Cr ratio density distribution**
28. Tafazoli et al. (2013)	Children; iADHD, cADHD	13/13	1.5 T, CSI, 1H-MRS (PRESS)	DLPFC re DLPFC li	NAA ↓, Cre ↓, t-Cho ↓, mI ↓ ↔
**29. Benamor (2014)**	**Children; hADHD, iADHD, cADHD**	**102 (57 medicated^a^, 45 unmedicated)/38**	**1.5 T, SVS, 1H-MRS (PRESS)**	**PFC le** **PFC ri** **Striatum le** **Striatum ri** **Cerebellum le**	**t-Cho/Cre** ↓ **in unmedicated ADHD (vs. medicated ADHD and controls)** ↔ **NAA/Cre** ↑ **in unmedicated ADHD (vs. medicated ADHD and controls)** ↔ **Glx/Cre** ↑ **in unmedicated ADHD (vs. medicated ADHD and controls)**
30. Maltezos et al. (2014)	Adults; iADHD, cADHD	40 (16 medicated^b^, 24 unmedicated)/20	1.5 T, SVS, 1H-MRS (PRESS)	Basal ganglia le DLPFC le Medial parietal lobe le	Glx ↓, Cre ↓, NAA ↓ Cre ↓ ↔
31. Husarova et al. (2014)	Children; cADHD	21/0	1.5 T, SVS, 1H-MRS (PRESS); before and after MPH/AM	DLPFC le DLPFC ri Anterior semioval center le Anterior semioval center ri	NAA and NAA/Cre ↓ after AM t-Cho/Cre ↑ after AM Glx ↑ and Glx/Cre ↑ after MPH ↔
32. Wiguna et al. (2014)	Children; cADHD and iADHD	21/0	1.5 T, SVS, 1H-MRS; before and after MPH	Amygdala le Amygdala ri	Glu/Cre ↓ after MPH Glu/Cre ↓ after MPH

*Cre, creatine; t-Cho, phosphorylcholine + glycerylphosphorylcholine; Glu, glutamate; Glx, glutamate + glutamine; NAA, *N*-acetylaspartate; mI, myo-Inositol; le, left; ri, right; ↑, increase in ADHD; ↓, decrease in ADHD; ↔, no metabolite differences between groups; MPH, methylphenidate; AM, atomoxetine; iADHD, inattentive ADHD subtype; cADHD, combined ADHD subtype; hADHD, hyperactive ADHD subtype; PRESS, point-resolved spectroscopy; STEAM, stimulated echo acquisition method; T, Tesla; SVS, single-voxel spectroscopy; CSI, chemical shift imaging; 31-P-MRS, phosphorus magnetic resonance spectroscopy; 1H-MRS, proton magnetic resonance spectroscopy; DLPFC, dorsolateral prefrontal cortex; ACC, anterior cingulate cortex; PCC, posterior cingulate cortex; MCC, medial cingulate cortex; free-PME, freely mobile membrane phospholipid precursors; free-PDE, freely mobile membrane phospholipid breakdown products*.

*Studies measuring the anterior cingulate cortex or cerebellar regions are in bold type*.

*^a^MPH or amphetamine*.

*^b^MPH or dextro-amphetamine*.

### Rationale of our study

Based on the evidence available during the finalization of the study protocol, we hypothesized that we would find altered fronto-striatal Glx and NAA signals in adult patients with ADHD as indirect evidence of dopaminergic dysfunction. Following the initial ­measurements of the striatum, which had very poor data quality, we abstained from our initial intent to measure the striatum and instead focused on the anterior cingulate cortex (ACC) and cerebellum. We adopted this approach because we already had data – which at that time was unpublished – pointing to glutamatergic abnormalities in these volumes of interest (VOIs) with decreased Glu/Cre ratios in the right pregenual ACC and increased Glu/Cre ratios in the left cerebellar hemisphere (Perlov et al., 2007, 2009, 2010).

## Materials and Methods

As an integral part of the multicenter COMPAS study, the imaging study received approval from the leading ethics committee (Faculty of Medicine, Freiburg University, 217/06) and authorization from the relevant German authorities (EudraCT No.: 2006-000222-31). Prior to beginning the study, the trial was registered with Current Controlled Trials.^2^ The methods, including the study design, endpoints, patient enrollment, and characteristics of the clinical study sample, have been described in detail previously (Philipsen et al., 2010, 2014). The study protocol is published in German on the Internet (Philipsen, 2008). All participants gave written consent for their participation in the imaging project.

### Patient assessment

The patient assessment took place between January 2007 and August 2010. The diagnostic procedure has been described in detail in two previous papers (Philipsen et al., 2010, 2014). All patients were stimulant-free for at least 6 months prior to scanning. Table 2 provides an overview of the screening process and the details of the MRS substudy’s selection procedure. Inclusion and exclusion criteria for the ADHD group are listed in Supplemental Table 1. The MRS sample was restricted to two study centers, one at Freiburg and one at Mannheim, in order to avoid scanner variance by performing measurements with only one MRI scanner at the Freiburg center. For this study, 113 ACC spectra and 104 cerebellar spectra fulfilled all the subsequent quality criteria for inclusion in statistical calculations.

**Table 2:** **ADHD and healthy control collective and reasons for exclusion**.

	**1480 patients contacted for study participation**		**Healthy controls were recruited via announcements on the campus and assessed as presented in Supplemental Table 2**	
	↓ (→ 962 ineligible or not interested)			
	**518 patients assessed for eligibility**			
	↓ (→ 274 patients from Berlin, Würzburg, Homburg, Essen, and Mainz)			
	**244 patients from Freiburg and Mannheim**			
	↓ (→ 57 ineligible or not interested in MRI substudy)			
	**187 patients potentially eligible for MRI substudy**		**119 healthy controls potentially eligible for MRI study**	

**Reasons for further exclusion**	**Anterior cingulate cortex**	**Cerebellum**	**Anterior cingulate cortex**	**Cerebellum**

Missing, incomplete, or pathological psychometric documentation	8	8	18	18
Study participation canceled, consent withdrawal, or non-compliance^a^	14	14	0	0
Metal implant	8	8	0	0
Claustrophobia	9	9	0	0
Abortion of measurement	5	5	3	3
Different voxel position	0	12	0	2
Failure in measurement protocol, data transfer, or data analysis	12	11	6	7
Bad spectral quality	8	6	4	5
New diagnosis of neurocytoma	1	1	0	0
*Post hoc* information about exclusion criteria	9	9	6	6

	**113 high-quality ACC and 104 high-quality cerebellar spectra for statistical analysis**		**82 high-quality ACC and 78 high-quality cerebellar spectra for statistical analysis**	

*ACC, anterior cingulate cortex. ^a^Failure to show up on the measurement dates*.

### Assessment of control subjects

The healthy control group was recruited via public announcements. We took great care to exclude relevant neuropsychiatric conditions and to provide psychometric assessments of our control subjects. The assessment instruments and exclusion criteria are illustrated in Supplemental Table 2. We were able to keep 82 ACC and 78 cerebellum data sets that fulfilled all quality criteria.

### Matching procedure

We attempted to select controls such that the distributions of demographic variables in controls would resemble those in patients. However, because many data sets had to be excluded after the measurements for data quality reasons, the final control sample was not fully matched. We considered but ultimately decided against a secondary matching procedure, in which we could have selected a smaller control sample from the pool of available control data that would have been matched with respect to all demographic data. Instead, we decided to include all the control data that fulfilled the methodological quality criteria, and we accounted for statistically significant differences in demographic variables by including the respective factors as covariates in the linear model to compare patients with controls.

### Data acquisition and analysis

**Figure 1:**
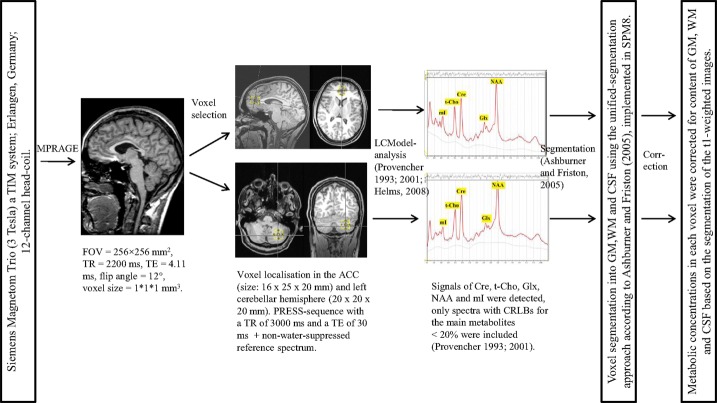
**MRI data acquisition and spectroscopic analyses**. MPRAGE, magnetization-prepared rapid-acquisition gradient echo; FOV, field of view; TR, repetition time; TE, echo time; ACC, anterior cingulate cortex; PRESS, point-resolved spectroscopy; LCModel, linear combination of model spectra; Cre, creatine; t-Cho, phosphorylcholine + glycerylphosphorylcholine; Glx, glutamate + glutamine; NAA, *N*-acetylaspartate; mI, myo-Inositol; ppm, parts per million; CRLBs, Cramér-Rao lower bounds; SPM8, Statistical Parametric Mapping – Version 8; GM, gray matter; WM, white matter; CSF, cerebrospinal fluid.

Magnetic resonance spectroscopy measurement and analysis were performed following a method established in previous studies (Tebartz van Elst et al., 2014a,b). All measurements were obtained at the University of Freiburg on a 3-Tesla whole-body scanner (Siemens Magnetom Trio a TIM system; Erlangen, Germany) using a 12-channel head-coil for signal reception. First, a T1-weighted 3D data set was recorded using a magnetization-prepared rapid-acquisition gradient echo (MPRAGE) sequence with the following parameters: field of view (FOV) = 256 mm × 256 mm, repetition time (TR) = 2200 ms, echo time (TE) = 4.11 ms, flip angle = 12°, and voxel size = 1 mm × 1 mm × 1 mm. For spectroscopic measurements, voxels were placed in the pregenual ACC (16 mm × 25 mm × 20 mm) and in the center of the left cerebellar hemisphere (20 mm × 20 mm × 20 mm). A point-resolved spectroscopy (PRESS) sequence with a TR of 3000 ms and a TE of 30 ms was used. For the absolute quantification of metabolites, we also acquired a non-water-suppressed reference spectrum. For spectroscopic analysis, the well-established LCModel (linear combination of model spectra) algorithm was used. The absolute quantification of metabolites (Cre, t-Cho, Glx, NAA, and mI) was estimated using an internal water signal reference (Provencher, 1993, 2001; Helms, 2008; Tebartz van Elst et al., 2014a,b). For further analyses, only spectra with Cramér-Rao lower bounds (CRLBs) for the main metabolites <20% were included (Provencher, 1993, 2001).^3^ To estimate the water content in the VOI, the MPRAGE was segmented into gray matter (GM), white matter (WM), and cerebrospinal fluid (CSF), using the unified-segmentation approach according to Ashburner and Friston (2005) and as implemented in Statistical Parametric Mapping, Version 8 (SPM8). For each spectroscopy voxel, the partial volumes of GM, WM, and CSF were computed from this segmentation. The metabolic concentrations of each VOI were correct for the contents of GM, WM, and CSF. Figure 1 summarizes all the details.

### Statistical analysis

Group comparisons for continuous variables (age, IQ, nicotine consumption, and psychometric scores) were performed with two-sided independent-sample *t*-tests. Group comparisons for gender were calculated using Pearson’s two-sided chi-squared test. For primary analysis, the neurometabolite concentrations of the patient and control groups were compared using a multivariate analysis of covariance and employing a general linear model (MANCOVA). The concentrations of neurometabolites were chosen as dependent variables. Of the possible influencing factors considered – age (Kaiser et al., 2005), gender, IQ (Jung et al., 1999), and nicotine consumption (Domino, 2008) – all except gender differed significantly between the ADHD and control groups (*p* < 0.05). Therefore, these values were included as covariates. To test for an overall group effect across all five neurometabolites, a multivariate Wilks’ lambda test was performed. Group differences per single neurometabolite concentration were tested and estimated with the confidence interval from the linear MANCOVA model, adjusting for the imbalanced influencing factors. Next, the ADHD subtypes were compared with the control groups using the same MANCOVA approach and employing a multivariate Wilks’ lambda test. Correlation analyses were performed using the Spearman correlation coefficient. For overall and single-group differences in neurometabolite concentrations, the level of significance was corrected for multiple tests using the Bonferroni approach (*p* < 0.025, because we measured two regions; 97.5% confidence intervals). For all subgroup and correlation analyses, we did not correct for multiple comparisons (*p* < 0.05 as the criterion of significance).

## Results

### Demographic and psychometric data

Table 3 summarizes the demographic and psychometric data for the ADHD and control groups following the exclusion of all compromised data sets. In both the ACC and the cerebellum samples, the patient and control groups significantly differed in age, IQ, and nicotine consumption, but not in gender. As expected, the psychometric scores for ADHD symptoms and depressiveness were significantly higher in the patient group.

**Table 3:** **Demographic and psychometric data**.

	Anterior cingulate cortex			Cerebellum		
	ADHD (*n* **=** 113)	Controls (*n* **=** 82)	*p*-value^a^	ADHD (*n* **=** 104)	Controls (*n* **=** 78)	*p*-value^a^
		
	Mean **±** SD/*n*	Mean **±** SD/*n*		Mean **±** SD/*n*	Mean **±** SD/*n*
Age	33.9 ± 9.9	36.8 ± 10.1	0.05	34.2 ± 10.2	37.8 ± 10.2	0.02
Gender	57M:56F	40M:42F	0.82	52M:52F	39M:39F	1.0
Intelligence quotient^b^	113.5 ± 15.7	120.2 ± 16.8	0.005	113.4 ± 16.0	119.6 ± 16.9	0.01
Nicotine (cigarettes/day)	5.7 ± 10.6	1.4 ± 3.8	<0.001	5.5 ± 9.8	1.4 ± 3.9	<0.001
Wender Utah Rating Scale (Retz-Junginger et al., 2002)	40.2 ± 8.8	8.4 ± 7.1	<0.001	40.3 ± 8.8	8.2 ± 7.2	<0.001
CAARS-ADHD-Index (Conners, 1999)	66.8 ± 11.9	39.5 ± 7.0	<0.001	66.7 ± 12.2	39.4 ± 6.7	<0.001
Beck Depression Inventory score (Hautzinger, 2006)	11.9 ± 8.0	2.3 ± 3.3	<0.001	11.4 ± 8.1	2.5 ± 3.4	<0.001
ADHD subtype	54 iADHD, 4 hADHD,^c^ 55 cADHD	No		47 iADHD, 4 hADHD,^c^ 53 cADHD	No	

*SD, standard deviation; M, male; F, female; CAARS, Conners Adult ADHD Rating Scales – self report: long version; iADHD, inattentive subtype; hADHD, hyperactive-impulsive subtype; cADHD, combined subtype*.

*^a^p-value: to test for differences between groups*.

*^b^Measured by the Multiple-Choice Vocabulary Intelligence Test (Lehrl et al., 1995)*.

*^c^The hyperactive subtype was added to the combined subtype for all subtype analyses because of the small group size*.

### MRS results

**Figure 2:**
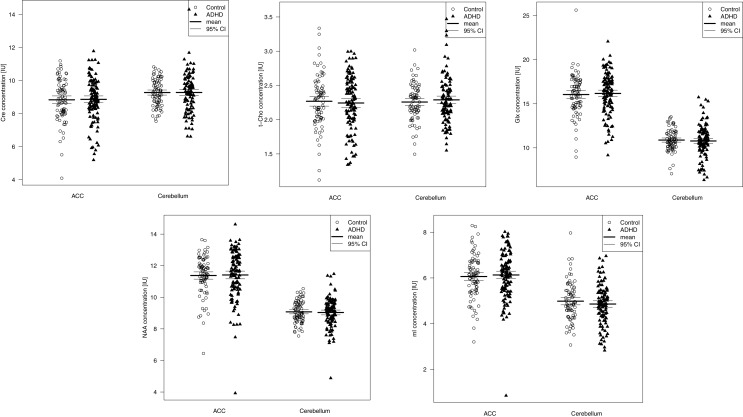
**Metabolite signals presented as scatterplots**. CI, 95% confidence interval; Cre, creatine; t-Cho, phosphorylcholine +glycerylphosphorylcholine; Glx, glutamate + glutamine; NAA, *N*-acetylaspartate; mI, myo-Inositol; IU, institutional unit.

Table 4 summarizes the spectroscopic results. Scatterplots of all individual metabolite concentrations, with means and 95% confidence intervals, are presented in Figure 2. Subgroup analyses are presented in Table 5 and correlation analyses are presented in Supplemental Table 3.

**Table 4:** **Spectroscopic findings in the pregenual ACC and the cerebellum (IU)**.

	Anterior cingulate cortex				Cerebellum			
	ADHD (*n* **=** 113) unadjusted mean **±** SD	Controls (*n* **=** 82) unadjusted mean **±** SD	Adjusted difference (97.5% CI)^a^	MANCOVA test for difference (Wilks’ lambda test: *p* **=** 0.97)^a^	ADHD (*n* **=** 104) unadjusted mean **±** SD	Controls (*n* **=** 78) unadjusted mean **±** SD	Adjusted difference (97.5% CI)^a^	MANCOVA test for difference (Wilks’ lambda test: *p* **=** 0.62)^a^
Cre	8.86 ± 1.32	8.84 ± 1.27	0.15 (−0.29 to 0.59)	*p* = 0.43	9.28 ± 1.17	9.28 ± 0.81	0.03 (−0.34 to 0.40)	*p* = 0.85
t-Cho	2.24 ± 0.40	2.27 ± 0.39	0.04 (−0.09 to 0.16)	*p* = 0.52	2.29 ± 0.34	2.26 ± 0.27	0.05 (−0.06 to 0.16)	*p* = 0.27
Glx	16.17 ± 2.37	16.05 ± 2.31	0.32 (−0.49 to 1.12)	*p* = 0.38	10.78 ± 1.84	10.88 ± 1.22	−0.24 (−0.81 to 0.33)	*p* = 0.35
NAA	11.41 ± 1.56	11.38 ± 1.24	0.17 (−0.33 to 0.66)	*p* = 0.44	9.04 ± 0.94	9.08 ± 0.68	−0.02 (−0.32 to 0.27)	*p* = 0.86
mI	6.13 ± 1.05	6.06 ± 0.96	0.10 (−0.25 to 0.45)	*p* = 0.51	4.86 ± 0.87	4.99 ± 0.85	−0.10 (−0.41 to 0.21)	*p* = 0.45

*SD, standard deviation; CI, confidence interval; Cre, creatine; t-Cho, phosphorylcholine + glycerylphosphorylcholine; Glx, glutamate + glutamine; NAA, *N*-acetylaspartate; mI, myo-Inositol*.

*^a^MANCOVA, multivariate analysis of covariance using the covariates age, IQ, and nicotine consumption*.

**Table 5:** **Subgroup analyses of spectroscopic findings in the anterior cingulate cortex and cerebellum of the inattentive and combined subtypes and controls (IU)**.

	iADHD (*n* = 54) unadjusted mean ± SD	Controls (*n* = 82) unadjusted mean ± SD	Adjusted difference (95% CI)^a^	MANCOVA test for difference (Wilks’ lambda test: *p* = 0.99)^a^	cADHD^b^ (*n* = 59) unadjusted mean ± SD	Controls (*n* = 82) unadjusted mean ± SD	Adjusted difference (95% CI)^a^	MANCOVA test for difference (Wilks’ lambda test: *p* = 0.86)^a^	
**Anterior cingulate cortex**								
Cre	8.73 ± 1.39	8.84 ± 1.27	0.10 (−0.38 to 0.58)	*p* = 0.68	8.99 ± 1.25	8.84 ± 1.27	0.23 (−0.21 to 0.68)	*p* = 0.31
t-Cho	2.18 ± 0.45	2.27 ± 0.39	0.01 (−0.14 to 0.15)	*p* = 0.91	2.30 ± 0.34	2.27 ± 0.39	0.08 (−0.05 to 0.20)	*p* = 0.22
Glx	16.04 ± 2.52	16.05 ± 2.31	0.30 (−0.59 to 1.18)	*p* = 0.51	16.28 ± 2.25	16.05 ± 2.31	0.47 (−0.34 to 1.29)	*p* = 0.25
NAA	11.32 ± 1.46	11.38 ± 1.24	0.12 (−0.37 to 0.61)	*p* = 0.64	11.50 ± 1.66	11.38 ± 1.24	0.23 (−0.29 to 0.74)	*p* = 0.38
mI	6.09 ± 0.94	6.06 ± 0.96	0.02 (−0.34 to 0.38)	*p* = 0.92	6.17 ± 1.16	6.06 ± 0.96	0.17 (−0.21 to 0.54)	*p* = 0.38

	**iADHD (*n* = 47) unadjusted mean ± SD**	**Controls (*n* = 78) unadjusted mean ± SD**	**Adjusted difference (95% CI)^a^**	**MANCOVA test for difference (Wilks’ lambda test: *p* = 0.71)^a^**	**cADHD^b^ (*n* = 57) unadjusted mean ± SD**	**Controls (*n* = 78) unadjusted mean ± SD**	**Adjusted difference (95% CI)^a^**	**MANCOVA test for difference (Wilks’ lambda test: *p* = 0.67)^a^**

**Cerebellum**								
Cre	9.21 ± 1.08	9.28 ± 0.81	0.06 (−0.30 to 0.42)	*p* = 0.74	9.34 ± 1.24	9.28 ± 0.81	0.02 (−0.35 to 0.39)	*p* = 0.91
t-Cho	2.28 ± 0.39	2.26 ± 0.27	0.07 (−0.06 to 0.19)	*p* = 0.30	2.30 ± 0.30	2.26 ± 0.27	0.04 (−0.06 to 0.4)	*p* = 0.42
Glx	10.88 ± 1.97	10.88 ± 1.22	−0.10 (−0.72 to 0.51)	*p* = 0.74	10.69 ± 1.74	10.88 ± 1.22	−0.30 (−0.83 to 0.23)	*p* = 0.27
NAA	8.98 ± 0.84	9.08 ± 0.68	−0.08 (−0.37 to 0.22)	*p* = 0.61	9.08 ± 1.01	9.08 ± 0.68	−0.01 (−0.32 to 0.29)	*p* = 0.93
mI	4.82 ± 0.84	4.99 ± 0.85	−0.14 (−0.48 to 0.20)	*p* = 0.42	4.89 ± 0.91	4.99 ± 0.85	−0.06 (−0.38 to 0.26)	*p* = 0.71

*SD, standard deviation; CI, confidence interval; iADHD, inattentive ADHD subtype; cADHD, combined ADHD subtype; Cre, creatine; t-Cho, phosphorylcholine + glycerylphosphorylcholine; Glx, glutamate + glutamine; NAA, *N*-acetylaspartate; mI, myo-Inositol*.

*^a^MANCOVA, multivariate analysis of covariance using the covariates age, IQ, and nicotine consumption*.

*^b^The hyperactive subtype was added to the combined subtype for all subtype analyses because of the small group size*.

#### Anterior Cingulate Cortex

A Wilks’ lambda test found no significant overall between-group group differences for the five neurometabolite concentrations in the ACC (*p* = 0.97). Furthermore, none of the metabolite concentrations were found to differ significantly between patients and controls. The inattentive and combined ADHD subtypes did not differ significantly from the control groups. A significant negative correlation between t-Cho and subclinical depression (measured via the Beck Depression Inventory score) was found (*p* = 0.0006).

#### Cerebellum

No overall metabolite differences between groups were found (Wilks’ lambda: *p* = 0.62) in the cerebellar VOI as well. Again, no differences in any of the five single metabolites were detected. Moreover, the subgroup analyses did not reveal any significant differences. The correlation analysis showed a discrete negative correlation between the mI signal and nicotine consumption (*p* = 0.04).

## Discussion

The main finding of this large study is the absence of any significant differences between patients and controls. We could not replicate earlier findings and could not confirm our working hypothesis regarding altered NAA and glutamate signals. In fact, we did not find evidence of any neurochemical abnormality.

### False-negative finding?

The issue of a potentially false-negative finding can be addressed by looking at the confidence intervals. Table 4 illustrates that all lower and upper bounds of the 97.5% confidence intervals for the estimated mean differences for all metabolites are no more than 0.6 standard deviations below or above 0. We can thus exclude even moderate differences between patients and controls corresponding to effect sizes of 0.6 and greater. This observation supports the notion that we have produced true negative results.

### Comparison to previous studies

How does this compare to previous research? We performed a comprehensive review of the MRS literature on ADHD (see Table 1). The respective results illustrate that our negative finding is in line with the majority of previous reports. While several earlier studies – including our own (Perlov et al., 2007, 2009, 2010) – did find significant differences in Glx signals and other neurometabolites, no clear pattern of signal change emerged across different studies.

What might be the reasons for these discrepant findings? Table 1 and Supplemental Table 4 illustrate that the sample sizes of several previous studies were rather small. Furthermore, differences in findings might be due partially to differences in scanning techniques, acquisition protocols, analytical algorithms, and sample-selection criteria. In previous studies, patient samples differed in terms of age, subtype, medication, and comorbidity.

Concerning age, studies on children and adults have to be distinguished because children have developing (i.e., dynamically changing) brains, whereas adult brains’ fundamental neurochemical and structural properties are not considered to change in a developmental manner. In the present study, we investigated only adult patients; therefore, developmental considerations should be negligible. Furthermore, the number of medicated patients differed in previous studies. In addition to being linked to short-term neurometabolic changes in some studies (Carrey et al., 2002; MacMaster et al., 2003; Wiguna et al., 2012; Husarova et al., 2014), MPH exposure may also cause long-term changes (Nakao et al., 2011; Frodl and Skokauskas, 2012). In our study, patients had gone at least 6 months without ADHD-specific medication. Therefore, it is unlikely that the normalizing effects of ADHD medication could explain our negative findings. The influence of comorbid diseases must also be taken into account because most psychiatric comorbidities might be related to changes in neurometabolism. In this study, relevant psychiatric comorbidities, such as present depressive disorder or other first-axis disorders, served as exclusion criteria. In summary, there is little evidence to suggest that age, medication, or the pathophysiology of comorbid neuropsychiatric disorders have contributed to our findings.

### Sample selection and MRS

This study was an integral part of the abovementioned COMPAS study. The sample selection followed strict standards (Philipsen et al., 2010, 2014). Only primary ADHD cases were included, and evidence of organic cerebral diseases led to exclusion. Therefore, our results cannot be generalized to patients with secondary forms of ADHD. We decided against secondary matching procedures and included all participants with good data quality in our statistical analyses. In doing so, we generated a large, carefully characterized, and fully transparent data set that minimized the risk of false-positive findings due, for example, to inadequate selection of controls. We methodically used the well-established and well-evaluated single-voxel proton spectroscopy to measure the absolute neurometabolite concentrations with an investigator-independent method (Tebartz van Elst et al., 2014a,b).

### Neurochemical perspective

Our data compel us to conclude that the brains of adult ADHD patients are essentially normal from a neurochemical perspective. Of course, we cannot generalize this finding to regions of the brain other than those that we measured. Furthermore, we cannot conclude that this proposition is true for children with ADHD. It might be the case that the reported abnormalities in children were true and that the respective neurochemical abnormalities linked to ADHD have normalized during maturation. However, this interpretation is not supported by our study because most of the clinical symptoms of ADHD were still present in our adult patient sample. Alternatively, in this scenario, one could conclude that the reported neurochemical abnormalities are not necessarily linked to the presence of the clinical ADHD symptoms. However, it is important to recognize that neurochemical processes measured by MRS are only a part of brain functioning. They are based on anatomical structures, which are organized in fronto-basal loops. The structural dysfunction of the fronto-striato-thalamo-frontal circuits plays a key role in the pathogenesis of ADHD (Perlov et al., 2009). These circuits have a common anatomical organization, beginning in the prefrontal cortex, innervating the striatum and the pallidum/substantia nigra, and ending in projections back in the frontal brain (Tekin and Cummings, 2002). For example, the ACC subcortical circuit plays an important role in motivation. The model of circuits illustrates that different neurochemical or anatomical lesions may lead to the same symptoms and that vice versa the same symptoms can be induced by different lesions (Tebartz van Elst and Perlov, 2013).

### Neuropsychiatric research perspective

The alternative interpretation involves a greater degree of skepticism about the scientific process as a whole. As in many other areas of neuropsychiatric research, initially promising findings cannot be replicated in large and methodologically sound studies. We reported relevant neurochemical findings regarding adult ADHD in both VOIs measured here. As illustrated in Table 1, the sample sizes of our recent studies compared well to those of other studies, and the methodology of these studies was also sound. However, we were not able to replicate our earlier findings – or those of others – in this large study. What can we conclude from this observation? First of all, it somewhat questions the notion that true abnormalities reported in children and adolescents might have normalized during the process of growing up, because in the area of adult research, we ourselves had reported significant findings in studies with a sample size of about 30 patients and controls that we are now unable to replicate (Perlov et al., 2007, 2010).

Altogether, the essentially negative findings of this large study support a more prudent approach to interpreting data from small studies. Recently, a series of papers addressed this problem in the context of biomedical research (Al-Shahi Salman et al., 2014; Chalmers et al., 2014; Chan et al., 2014; Glasziou et al., 2014; Ioannidis et al., 2014; Macleod et al., 2014). Particularly in imaging research, numerous studies have small and sometimes ill-defined patient samples. The issue of replication is often neglected. Positive findings from different studies are often used as evidence supporting the findings of the study of interest. However, the implicit negative results of many other studies are neglected. For example, we ourselves summarized all the previous MRS studies on ADHD (Table 1) and focused on their positive findings. However, every study that does not repeat the findings of previous studies must be regarded as a non-replication. The fact that most papers focus on positive findings blurs this view. Meta-analyses are one way to pool respective data. However, as in this case, they tend to generate negative findings, particularly if a cross-meta-analytical perspective is taken. Our first meta-analysis of MRS findings in ADHD showed an increase in only the t-Cho signal in the striatum and right frontal lobe of children and in the ACC of adult patients (Perlov et al., 2009). A recent meta-analysis described only an increased NAA signal in the medial prefrontal cortex of children and showed no abnormalities in the adult ADHD group (Aoki et al., 2013). From a cross-meta-analytical perspective, we have to conclude that no specific patterns of MRS abnormalities emerged upon observation of the results of both papers.

### The nosology problem

Another possible reason for a false-negative finding has to be considered at this point. ADHD is generally understood as a disorder of heterogeneous etiologies, which means that it is quite possible that as a result of generating large patient samples, different etiological ADHD subgroups will be examined within one study group. For practical reasons, it might well be the case that in generating large study samples, the likelihood of diversifying the study group increases from an etiological point of view. For example, when organizing large multicenter study groups, it is more likely that different diagnosticians will be involved in the study process even if the study protocol is, like ours, very prudent. For this reason, large studies might be more vulnerable to diverse underlying etiologies than studies with smaller samples generated by one or a few diagnosticians.

The issue of ADHD subforms and subtypes must also be considered. All possible secondary forms of ADHD were excluded from our study. Primary or secondary forms of ADHD and different ADHD subtypes might represent different pathophysiologies (this is comparable to autism spectrum disorder as a basic disorder) (Tebartz van Elst et al., 2013). We think that progress in neuropsychiatric research in general, and neurobiological ADHD research in particular, will be closely linked to the recognition of this nosology problem (Tebartz van Elst et al., 2006). If it is true that a purely clinically defined group of ADHD patients represents different etiologies and cerebral pathophysiologies, then the inclusion of different pathophysiologies within one study sample will necessarily lead to diverse and contradictory results in different samples (Tebartz van Elst et al., 2014a,b). The larger and more pathophysiologically heterogeneous a single study sample is, the more likely it is that true signal differences in subgroups of the sample will statistically counterbalance each other and result in normal average signals in calculations of the means of the overall group. Further studies and conceptual work will have to tackle this problem.

## Conclusion

To date, this is the largest MRS study examining cerebral neurochemistry in ADHD. We were able to demonstrate an essentially normal neurochemical profile of the ACC and the cerebellum in adult patients without current comorbid psychiatric disorders. We were unable to replicate earlier positive findings. Such previous positive findings might have been linked to small sample size, psychiatric comorbidity, or medication effects. However, the nosology problem of psychiatry (i.e., disorder categories comprise patient subgroups with different pathophysiologies) also has to be considered when interpreting the negative findings of large neuropsychiatric study samples.

## Conflict of Interest Statement

Peter Goll has received travel grants from GSK, Boston Scientific, and Otsuka Pharma. Esther Sobanski has received speakers’ honoraria from Medice, Eli Lilly, and Novartis; she is a member of the advisory boards of Medice, Shire, and Eli Lilly; and has performed phase III studies and IITs with Medice, Novartis, Janssen Cilag, and Eli Lilly. Alexandra Philipsen served on advisory boards, gave lectures, performed phase III studies, and received travel grants within the last three years from Eli Lilly, Janssen-Cilag, Medice Arzneimittel Pütter GmbH, Novartis, and Shire. Alexandra Philipsen is also the author of several books and articles on psychotherapy published by Elsevier, Hogrefe, Schattauer, Kohlhammer, and Karger. Ludger Tebartz van Elst served on advisory boards, gave lectures, and received travel grants within the last 3 years from Eli Lilly, Janssen-Cilag, Novartis, Shire, UCB, GSK, Servier, Janssen, and Cyberonics. The other authors declare that the research was conducted in the absence of any commercial or financial relationships that could be construed as potential conflicts of interest.

## Supplementary Material

The Supplementary Material for this article can be found online at http://journal.frontiersin.org/article/10.3389/fnbeh.2015.00242

## Funding

This study was funded by the German Federal Ministry of Science and Education (BMBF) (ADHD-NET: 01GV0605, 01GV0606).

## Footnotes

^1^www.dsm5.org

^2^www.isrctn.com/ISRCTN54096201

^3^www.s-provencher.com/pages/lcmodel.shtml
