# Supplementary material for: Normal Neurochemistry in the Prefrontal and Cerebellar Brain of Adults with Attention-Deficit Hyperactivity Disorder
Source: Front Behav Neurosci. 2015 Sep 28;9:242. doi: 10.3389/fnbeh.2015.00242 (PMC4585345; doi:10.3389/fnbeh.2015.00242)
Supplement: Supplementary file 1 [file Data_Sheet_1.PDF]

# Supplementary Information

**SUPPLEMENTAL TABLE 1: Inclusion and exclusion criteria (Philipsen, 2008).**

**Inclusion criteria**

- Male and female
- Subjects must speak German fluently
- Aged 18–60 years inclusive
- Diagnosis of ADHD according to the *DSM-IV* criteria (http://www.dsm5.org)
- A score of > 30 on the short version of the Wender Utah Rating Scale (Retz-Junginger et al., 2002) or a clinically assured ADHD diagnosis in childhood
- Chronic course of ADHD symptoms from childhood to adulthood
- Subjects provided written informed consent in accordance with international guidelines and local legislation
- Unobtrusive physical examination (including blood pressure/heart rate) without serious or uncontrolled findings
- Lab results without clinically relevant findings (e.g., blood count, renal retention data, tests of liver function, thyroid parameters)
- EKG and EEG without pathologically relevant results

**Exclusion criteria**

- IQ < 85 according to a score of < 17 on the Multiple-Choice Vocabulary Intelligence Test (German version; Lehrl et al., 1995)
- Schizophrenia, bipolar affective disorder, borderline personality disorder, antisocial personality disorder, suicidality or self-harm, autism, motor tics, or Tourette syndrome
- Current eating disorder (bulimia nervosa, anorexia nervosa, body mass index < 19)
- Substance abuse or dependence in the 6 months previous to the screening. Episodic consumption is not an exclusion criterion. A positive drug test during screening.
- Neurological disorders, seizures, pathological EEG results (lateral differences, lesion, epileptiform potentials), glaucoma, diabetes mellitus, fasting blood glucose level > 110 mg/dl, hyperlipidemia, uncontrolled arterial hypertension (according to the guidelines of the German Hypertension Society), angina pectoris, known arterial occlusive disease or other manifestation of vascular disease, known tachycardic arrhythmias, or known enlarged prostate
- History of stroke
- Medication with stimulants or ADHD-specific psychotherapy in the 6 months previous to the beginning of the study
- Unwillingness or inability to comply with the requirements of the study protocol
- Inability to understand the nature, significance, and scope of the study

ADHD, attention-deficit hyperactivity disorder; DSM-IV, Diagnostic and Statistical Manual of Mental Disorders - Fourth Edition; EKG, electrocardiogram; EEG, electroencephalography; IQ, intelligence quotient.

**SUPPLEMENTAL TABLE 2: Assessment instruments and exclusion criteria for the control group.**

| **Diagnostic instrument** | **Instrument description** | **Exclusion criteria** |
|---|---|---|
| Semistructured interview | Demographics, psychosocial, and medical history | - Any current or previous psychiatric or neurological disease<br>- The consumption of any psychotropic drugs |
| Multiple-Choice Vocabulary Intelligence Test (MWT-B) (Lehrl et al., 1995) | Screening for verbal intelligence quotient | - Intelligence quotient < 85 according to a score of < 17 |
| Mini International Neuropsychiatric Interview (M.I.N.I.) (Sheehan et al., 1998) | Axis-I disorders | - Any axis-I disorders |
| Conners Adult ADHD Rating Scale (CAARS-S:L) (Conners, 1999) | Current ADHD symptoms | - CAARS t-scores for all subscores above 65 |
| Wender-Utah-Rating-Scale (WURS-k) (Retz-Junginger et al., 2002) | Childhood ADHD symptoms | - Scores above 30 |
| Beck Depression Inventory (BDI) (Hautzinger, 2006) | Depressive symptoms | - Scores above 18 |

ADHD, attention-deficit hyperactivity disorder.

**SUPPLEMENTAL TABLE 3: Spearman correlation analyses in patient sample (anterior cingulate cortex: n = 113; cerebellum: n = 104).**

| | **Anterior cingulate cortex** | | | |
|---|---|---|---|---|
| | **WURS** (Retz-Junginger et al., 2002) | **CAARS-ADHD-Index** (Conners, 1999) | **BDI** (Hautzinger, 2006) | **Nicotine** (cigarettes/day) |
| Cre | -0.06 | -0.04 | -0.15 | -0.08 |
| | 0.55 | 0.68 | 0.12 | 0.38 |
| t-Cho | -0.06 | -0.12 | **-0.32** | -0.09 |
| | 0.55 | 0.21 | **0.0006** | 0.33 |
| Glx | -0.03 | -0.03 | -0.09 | -0.07 |
| | 0.77 | 0.72 | 0.37 | 0.49 |
| NAA | -0.06 | 0.09 | -0.13 | -0.12 |
| | 0.55 | 0.36 | 0.17 | 0.21 |
| mI | 0.04 | -0.07 | -0.16 | 0.08 |
| | 0.67 | 0.48 | 0.10 | 0.41 |
| | **Cerebellum** | | | |
| | **WURS** (Retz-Junginger et al., 2002) | **CAARS-ADHD-Index** (Conners, 1999) | **BDI** (Hautzinger, 2006) | **Nicotine** (cigarettes/day) |
| Cre | -0.18 | 0.03 | -0.07 | -0.04 |
| | 0.07 | 0.78 | 0.46 | 0.66 |
| t-Cho | -0.03 | 0.09 | -0.05 | -0.09 |
| | 0.77 | 0.36 | 0.59 | 0.37 |
| Glx | 0.02 | 0.01 | 0.00 | 0.05 |
| | 0.81 | 0.95 | 0.97 | 0.64 |
| NAA | -0.12 | 0.08 | -0.12 | 0.060 |
| | 0.24 | 0.41 | 0.23 | 0.56 |
| mI | -0.01 | 0.06 | 0.03 | **-0.21** |
| | 0.93 | 0.56 | 0.77 | **0.04** |

Data are Spearman correlation coefficients and p-values. Cre, creatine; t-Cho, phosphorylcholine + glycerylphosphorylcholine; Glx, glutamate + glutamine; NAA, N-acetylaspartate; mI, myo-Inositol; WURS-k, Wender Utah Rating Scale; CAARS, Conners Adult ADHD Rating Scales - Self Report: Long Version; BDI, Beck Depression Inventory score.

**SUPPLEMENTAL TABLE 4: MRS studies on the anterior cingulate cortex and cerebellum.**

| Study | Results | Methodology | Localization | Subtype | Gender | Age | Medication |
|---|---|---|---|---|---|---|---|
| **Anterior cingulate cortex** | | | | | | | |
| Moore et al. (2006)<br>(n = 23 ADHD patients/7 controls) | Glx/mI↑ | 1,5 T, SVS, 1H-MRS (STEAM) | ACC both sides (2x2x1,2 cm) | Unclear | Unclear | Children, adolescents | 3 patients were medicated |
| Perlov et al. (2007)<br>(n = 28/28) | le: ↔<br>ri: Glx/Cre↓ | 1,5 T, CSI, 1H-MRS (PRESS) | ACC ri+le | Unclear | Almost balanced | Adults | No medication for minimum 6 months |
| Colla et al. (2008)<br>(n = 15/10) | le: ↔<br>ri: t-Cho↑ (Glx not measured) | 1,5 T, CSI, 1H-MRS (PRESS) | ACC ri+le | Unclear | Almost balanced | Adults | Naive |
| Kronenberg et al. (2008)<br>(n = 7/0) | t-Cho↓, NAA↑ with MPH | 1,5 T, CSI, 1H-MRS (PRESS); before and after MPH | ACC ri+le | Unclear | Male > female | Adults | Naive, then 5–6 weeks MPH |
| Hammerness et al. (2012)<br>(n = 10/12) | ↔ | 4 T, SVS, 1H-MRS (PRESS); before and after MPH | ACC both sides (2x2x2 cm) | Unclear | Mainly boys | Adolescents | Stopped 1–4 weeks before (n = 8), naive (n = 2) |
| Arcos-Burgos et al. (2012)<br>(n = 14/20) | ↔ | 1,5 T, CSI, 1H-MRS (PRESS) | ACC ri+le | Unclear | Female > male | 11 adults, 2 children | Naive |
| **Cerebellum** | | | | | | | |
| Soliva et al. (2010)<br>(n = 17/17) | mI↓, NAA↓, Cre↓ | 1,5 T, SVS, 1H-MRS (PRESS) | Cerebellum le (2×2×2 cm) | Mainly cADHD | Mainly boys | Children | All medicated |
| Perlov et al. (2010)<br>(n = 30/30) | le: Glx/Cre↓<br>ri: ↔<br>Vermis: ↔ | 1,5 T, CSI, 1H-MRS (PRESS) | Cerebellum (le+ri+vermis) | Only cADHD | Almost balanced | Adults | No medication for minimum 6 months |
| BenAmor (2014)<br>(n = 102/38) | Glx/Cre ↑ in unmedicated ADHD (vs. medicated ADHD and controls) | 1,5 T, SVS, 1H-MRS (PRESS) | Cerebellum le (8.0 $cm^3$) | Mainly cADHD, also iADHD, hADHD | Mainly boys | Children | 45 naive, 57 used stimulants |

Cre, creatine; t-Cho, phosphorylcholine + glycerylphosphorylcholine; Glx, glutamate + glutamine; NAA, N-acetylaspartate; mI, myo-Inositol; le, left; ri, right; ↑, increased in ADHD; ↓, decreased in ADHD; ↔, no metabolite differences between groups; MPH, methylphenidate; iADHD, inattentive ADHD subtype; cADHD, combined ADHD subtype; hADHD, hyperactive ADHD subtype; PRESS, point-resolved spectroscopy; STEAM, stimulated echo acquisition method; T, Tesla; SVS, single-voxel spectroscopy; CSI, chemical shift imaging.

# References for Supplementary Information


Arcos-Burgos, M., Londoño, A. C., Pineda, D. A., Lopera, F., Palacio, J. D., Arbelaez, A., Acosta, M. T., Vélez, J. I., Castellanos, F. X., & Muenke, M. (2012). Analysis of brain metabolism by proton magnetic resonance spectroscopy ((1)H-MRS) in attention-deficit/hyperactivity disorder suggests a generalized differential ontogenic pattern from controls. *Attention deficit and hyperactivity disorders*, *4*, 205–212. doi:10.1007/s12402-012-0088-0

Benamor, L. (2014). (1)H-Magnetic resonance spectroscopy study of stimulant medication effect on brain metabolites in French Canadian children with attention deficit hyperactivity disorder. *Neuropsychiatric disease and treatment*, *10*, 47–54. doi:10.2147/NDT.S52338

Colla, M., Ende, G., Alm, B., Deuschle, M., Heuser, I., & Kronenberg, G. (2008). Cognitive MR spectroscopy of anterior cingulate cortex in ADHD: elevated choline signal correlates with slowed hit reaction times. *Journal of psychiatric research*, *42*, 587–595. doi:10.1016/j.jpsychires.2007.06.006

Conners, C. K. (1999). *Conners' Adult ADHD Rating Scales (CAARS)*. North Tonawanda, NY: Multi-Health Systems.

Hammerness, P., Biederman, J., Petty, C., Henin, A., & Moore, C. M. (2012). Brain Biochemical Effects of Methylphenidate Treatment Using Proton Magnetic Spectroscopy in Youth with Attention-Deficit Hyperactivity Disorder: A Controlled Pilot Study. *CNS neuroscience & therapeutics*, 18:34-40. doi:10.1111/j.1755-5949.2010.00226.x

Hautzinger, M. (2006). *Das Beck Depressionsinventar II. Deutsche Bearbeitung und Handbuch zum BDI II*. Frankfurt am Main: Harcourt Test Services.

Kronenberg, G., Ende, G., Alm, B., Deuschle, M., Heuser, I., & Colla, M. (2008). Increased NAA and reduced choline levels in the anterior cingulum following chronic methylphenidate. A spectroscopic test-retest study in adult ADHD. *European archives of psychiatry and clinical neuroscience*, *258*, 446–450. doi:10.1007/ s00406-008-0810-2

Lehrl, S., Triebig, G., and Fischer, B. (1995). Multiple choice vocabulary test MWT as a valid and short test to estimate premorbid intelligence. *Acta Neurol. Scand.* 91, 335–345. doi:10.1111/j.1600-0404.1995.tb07018.x

Moore, C. M., Biederman, J., Wozniak, J., Mick, E., Aleardi, M., Wardrop, M., Dougherty, M., Harpold, T., Hammerness, P., Randall, E., & Renshaw, P. F. (2006). Differences in brain chemistry in children and adolescents with attention deficit hyperactivity disorder with and without comorbid bipolar disorder: a proton magnetic resonance spectroscopy study. *The American journal of psychiatry*, *163*, 316–318. doi:10.1176/appi.ajp.163.2.316

Perlov, E., Philipsen, A., Hesslinger, B., Buechert, M., Ahrendts, J., Feige, B., Bubl, E., Hennig, J., Ebert, D., & van Tebartz Elst, L. (2007). Reduced cingulate glutamate/glutamine-to-creatine ratios in adult patients with attention deficit/hyperactivity disorder -- a magnet resonance spectroscopy study. *Journal of psychiatric research*, *41*, 934–941. doi:10.1016/j. jpsychires.2006.12.007

Perlov, E., van Tebarzt Elst, L., Buechert, M., Maier, S., Matthies, S., Ebert, D., Hesslinger, B., & Philipsen, A. (2010). $H^1$-MR-spectroscopy of cerebellum in adult attention deficit/hyperactivity disorder. *Journal of psychiatric research*, *44*, 938–943. doi:10.1016/j.jpsychires.2010.02.016

Philipsen A. (2008). P R Ü F P L A N: Vergleich einer strukturierten störungsspezifischen Gruppenpsychotherapie plus Placebo oder Methylphenidat versus einer psychiatrischen Beratung plus Placebo oder Methylphenidat bei ADHS im Erwachsenenalter – eine erste randomisierte Multizenter-Studie (BMBF-ADHD-C1). http://www.uniklinik-freiburg.de/fileadmin/mediapool/07_kliniken/psy_psychiatrie/pdf/forschung/Pr%C3%BCfplan_Protokollversion_5_-Amendment-4_17.12.08.pdf.

Retz-Junginger, P., Retz, W., Blocher, D., Weijers, H. G., Trott, G. E., Wender, P. H., & Rössler, M. (2002). Wender Utah Rating Scale (WURS-k) Die deutsche Kurzform zur retrospektiven Erfassung des hyperkinetischen Syndroms bei Erwachsenen. *Der Nervenarzt*, *73*, 830–838. doi:10.1007/s00115-001-1215-x

Sheehan, D. V., Lecrubier, Y., Sheehan, K. H., Amorim, P., Janavs, J., Weiller, E., Hergueta, T., Baker, R., & Dunbar, G. C. (1998). The Mini-International Neuropsychiatric Interview (M.I.N.I.): the development and validation of a structured diagnostic psychiatric interview for DSM-IV and ICD-10. *The Journal of clinical psychiatry*, *59 Suppl 20*, 22-33; quiz 34-57.

Soliva, J. C., Moreno, A., Fauquet, J., Bielsa, A., Carmona, S., Gispert, J. D., Rovira, M., Bulbena, A., & Vilarroya, O. (2010). Cerebellar neurometabolite abnormalities in pediatric attention/deficit hyperactivity disorder: a proton MR spectroscopic study. *Neuroscience letters*, *470*, 60–64. doi:10.1016/j.neulet.2009.12.056
